# SLC12A8 plays a key role in bladder cancer progression and EMT

**DOI:** 10.1515/med-2021-0013

**Published:** 2020-12-08

**Authors:** Shun-Lai Li, Zheng-Feng Li, Qing-Wei Cao, Wen-Zhen Wang

**Affiliations:** The Fifth People’s Hospital of Jinan, Department of Urology, No. 24297, Jingshi Road, Huaiyin District, Jinan, Shandong, China; Shandong Provincial Hospital, Department of Urology, No. 9677, Jingshi Road, Lixia District, Jinan, Shandon, China

**Keywords:** SLC12A8, bladder cancer, upregulation, EMT, prognosis

## Abstract

Bladder cancer is the most common malignant tumor of the urinary system. The intention of the present research is to explore the prognostic value and biological function of solute carrier family 12 member 8 (SLC12A8) in bladder cancer. The analysis based on the TCGA and ONCOMINE database revealed that the expression of SLC12A8 in bladder cancer was notably increased compared with the normal group. SLC12A8 expression was notably correlated with the age, pathological stage, T-stage, and lymph node metastasis of bladder cancer patients. Moreover, the patients’ overall survival was notably shorter in the high SLC12A8 group. Compared with the control, SLC12A8 upregulation enhanced the proliferative, invasive, and migratory capacities of bladder cancer cells and promoted the expression of epithelial–mesenchymal transition (EMT) protein markers including β-catenin, vimentin, snail, and slug, while reduced the expression of E-cadherin. In the case of downregulated SLC12A8 expression, the proliferative, invasive, and migratory capacities of bladder cancer cells and the expression of EMT protein markers presented the opposite trend. This study demonstrated that SLC12A8 was highly correlated with oncogenesis and progression of bladder cancer, indicating that SLC12A8 may be a meaningful biomarker for initial diagnosis and early treatment of bladder cancer.

## 1 Introduction

Bladder cancer, one of the leading causes of death worldwide, is the most common malignant tumor of the urinary system. The incidence rate of this disease is increasing year by year [1]. Due to the shortage of predictive indicators, most bladder cancer patients could be diagnosed only in the middle or advanced stage. Despite significant advances in surgical treatment, chemotherapy technology, and radiation therapy, the overall survival (OS) of patients with bladder cancer still remains poor [2]. The molecular mechanism of bladder cancer proliferation, invasion, and metastasis is one of the hot spots in the study of oncogenesis, development, and treatment [3]. Revealing specific molecular pathogenesis is vital to the development of more effective diagnoses and treatments of bladder cancer.

Solute carrier family 12 member 8 (SLC12A8), as a nicotinamide mononucleotide transporter, belongs to the SLC12 gene family of the electroneutral cation–chloride-coupled cotransporters [4,5,6]. The SLC12 gene family is widely found in various human tissues, and it is the molecular basis for maintaining the balance of liquid metabolism in the body [7]. The family has two major branches: one is a sodium-dependent cotransporter, including SLC12A2, SLC12A1, and SLC12A3, and the second is a potassium and chloride cotransporter containing four members (SLC12A4, SLC12A5, SLC12A6, and SLC12A7); besides, there are also two other members, SLC12A8 and SLC12A9, whose functions have not been revealed [8,9]. From the current researches on SLC12 family, we learned that the expression of SLC12A1 is associated with glioma and hepatocellular carcinomas [10,11]. Moreover, it has been reported that the overexpression of SLC12A5 promoted the development and metastasis of tumor and is highly related to the significant decrease in patients’ survival with colorectal carcinoma and ovarian carcinoma [12,13]. In addition, SLC12A7 was found to be correlated with adrenocortical carcinoma by altering the adhesion properties of cancer cells [14,15,16]. However, the study on SLC12A8 remains limited up to now. On the basis of current researches, we found that the upregulation of SLC12A8 was related to psoriasis, psoriatic arthritis, atopic dermatitis, and breast cancer [17,18,19]. But to our knowledge, no report has been published about the biological function and the regulation of SLC12A8 expression in bladder cancer.

Therefore, we investigated the biological effects of SLC12A8 on the bladder cancer cells and the association between SLC12A8 expression and prognosis of patients with this disease, which will provide a basis for clarifying the mechanism of bladder cancer metastasis and early diagnosis for this disease.

## 2 Materials and methods

### 2.1 Bioinformatics analysis

The RNASeq data from TCGA (https://cancergenome.nih.gov/) [20], including 414 samples of bladder cancer patients and 19 normal samples, were used for the SLC12A8 expression analysis. Among the 414 tumor samples, 405 cases with relatively complete clinical data were used to study the relationship between SLC12A8 expression and clinical factors. Moreover, the data obtained from TCGA and GTEx (tumor = 411, normal = 28) were processed by UCSC (https://genome.ucsc.edu/) and used for the SLC12A8 expression analysis. In addition, the SLC12A8 expression in infiltrating bladder urothelial carcinoma and normal tissues was analyzed using Oncomine database (https://www.oncomine.org). The median expression of the gene was used as a boundary to classify the samples into high and low expression groups. Gene expression profiling interactive analysis (GEPIA; http://gepia.cancer-pku.cn/) website was applied to detect the relationship between SLC12A8 and E-cadherin/β-catenin/vimentin/snail/slug in bladder cancer by the Pearson correlation coefficient.

### 2.2 Cell culture

The human bladder cancer cell lines (5637, J82, and T24) and normal control cells SV40-immortalized human uroepithelial cells (SV-HUC-1) were obtained from the Chinese Academy of Medical Sciences (Shanghai, Chinese). All cells were cultured in RPMI-1640 mixed with 100 units/mL penicillin, 100 µg/mL streptomycin, and 10% fetal bovine serum. Then, they were maintained under 5% CO_2_ at 37°C for subsequent experiments.

### 2.3 Transfection

The T24 cell line underwent transfection with si-SLC12A8#1 (5′-GCCATGTATATCACCGGCTT-3′) or si-SLC12A8#2 (5′-CCTACAAGATAGCTTCCTCT-3′). The control siRNA sequence (si-con) was 5′-CGAACUCACUGGUCUGACC-3′. For upregulation of SLC12A8, the plasmid vector pcDNA3.1-SLC12A8 was transfected into 5637 cell line, and pcDNA3.1 empty vector was used as a control. Transfection was performed as described in the Lipofectamine 2000 Transfection Kit. When the cell concentration reached 80% in the six-well plate, the antibiotic-free medium was replaced 2 h before the transfection, and then, cell transfection was performed. The expression of the SLC12A8 could be observed after 24 h.

### 2.4 RNA isolation and qRT-PCR assays

The total RNA was extracted by Trizol reagent. After reverse transcription to form cDNA, the real-time PCR system was performed to assess mRNA-SLC12A8 expression. The qRT-PCR condition was as follows: initial denaturation (5 min, 95°C), 40 cycles of denaturation (30 s, 95°C), annealing (45 s, 60°C), and extension (30 s, 72°C). Each experiment was assessed in triplicate. Quantification was done using the 2^−ΔΔCT^ method to calculate the mRNA expression level of SLC12A8. The sequences of primers in this assay were listed as follows: SLC12A8: F: 5′- TGGCGTCTACTCCATGATCTCC-3′, R: 5′-CCGAGATGGATTCAGCAAAGCC-3′; GAPDH: F: 5′-TGTGTCCGTCGTGGATCTGA-3′, R: 5′-CCTGCTTCACCACCTTCTTGA-3′. GAPDH served as the internal reference.

### 2.5 Western blotting assays

After transfection for 24 h, cells in the six-well plate were placed on ice. After extracting by RIPA lysate (with protease inhibitor), the concentrations of protein were determined by the BCA method. Then, proteins were heated at 95°C for 5 min before isolating by SDS–PAGE, and they were transferred onto a PVDF membrane subsequently. The membrane was then blocked for 1 h using 5% skim milk at an ambient temperature. Following that, the membrane was incubated with primary antibodies at 4°C overnight. The primary antibodies used in this study were as follows: SLC12A8 (ab122152, Abcam), E-cadherin (ab40772, Abcam), β-catenin (ab227499, Abcam), vimentin (ab45939, Abcam), snail (ab82846, Abcam), and slug (ab27568, Abcam). After incubation, the membrane was taken out and washed using TBST (tris buffered saline with Tween) for 3 × 5 min. Subsequently, bands were finally examined by ECL (enhanced chemiluminescence) after incubating the membrane with the secondary antibody at an ambient temperature for 1 h. The gray values were scanned by Quantity One software, and the ratio of target protein/GAPDH was calculated.

### 2.6 Cell viability assays

Cell viability was detected by the CCK8 kit (Lianke Bio, China). Briefly, the cells were digested after 24 h transfection to prepare cell suspension. Then, cell suspension was plated in 96-well plates, and the number of cells in each well was about 1 × 10^3^. The 96-well plates were incubated under standard conditions, and cell viability was examined every 24 h. A total of 10 µL CCK8 was added into each well at the indicated time points and incubated for 1.5 h at 37°C. Finally, the optical density (OD) value at 450 nm was detected, and the proliferation curve was drawn.

### 2.7 Transwell invasion and migration assays

After transfection, cells were treated to prepare cell suspension with serum-free culture. Then, cells were inoculated in the upper chamber at the density of 1 × 10^5^ cells per well. The upper layer membranes were either coated with or without 100 µL Matrigel (serum-free medium diluted 1:6), which used to evaluate the migration and the invasion capacity respectively. About 500 µL culture medium was placed in the bottom chambers. Cells were grown in six-well plates overnight, and then, the chambers were taken out. The cells on the upper layers were wiped by swab, and the cells that invaded and migrated to the bottom layer were fixed in 4% paraformaldehyde for 0.5 h and stained using 0.1% crystal violet for 20 min after washing with PBS. Cells in five different fields were selected for calculation, and images were captured under a microscope.

### 2.8 Plate clone formation assay

Exponentially growing cells were trypsinized to individual cells to make cell suspensions. Then, cells were seeded in 60 mm Petri dish at 400 cells/Petri dish density with 5 mL culture solution prewarmed at 37°C. After that, cells were incubated under 5% CO_2_ at 37°C for 1–2 weeks to form colonies. The cell culture was terminated when there is visible clone formation. Subsequently, cells were stained using 0.1% crystal violet for 0.5 h after being fixed with 5 mL 4% paraformaldehyde for 0.5 h. The Petri dish was air dried after the staining solution was slowly washed with the running water. Then, the number of clones was counted and the size of clones were compared.

### 2.9 Data analysis and statistics

SPSS 22.0 and Graphpad Prism 5.0 software were performed for all the statistical analyses, and all experiments data were represented as means ± SD. The comparison between two composition ratios of the data was based on Student’s *t*-test, and comparison among multiple samples was conducted by one-way ANOVA with post hoc Dunnett’s test [21]. Kaplan–Meier analysis was conducted in SPSS 22.0 software to evaluate the association between SLC12A8 expression and survival of bladder cancer patients. Furthermore, the log-rank test was adopted to evaluate the statistical significance [22]. To investigate whether SLC12A8 can be used as an independent predictor of bladder cancer prognosis, Cox regression was used. The association between gene expression and clinical features was assessed using the chi-square test. *p* < 0.05 was regarded as significance, and all experiments were repeated for three times.

## 3 Results

### 3.1 SLC12A8 was upregulated in bladder cancer

**Figure 1:**
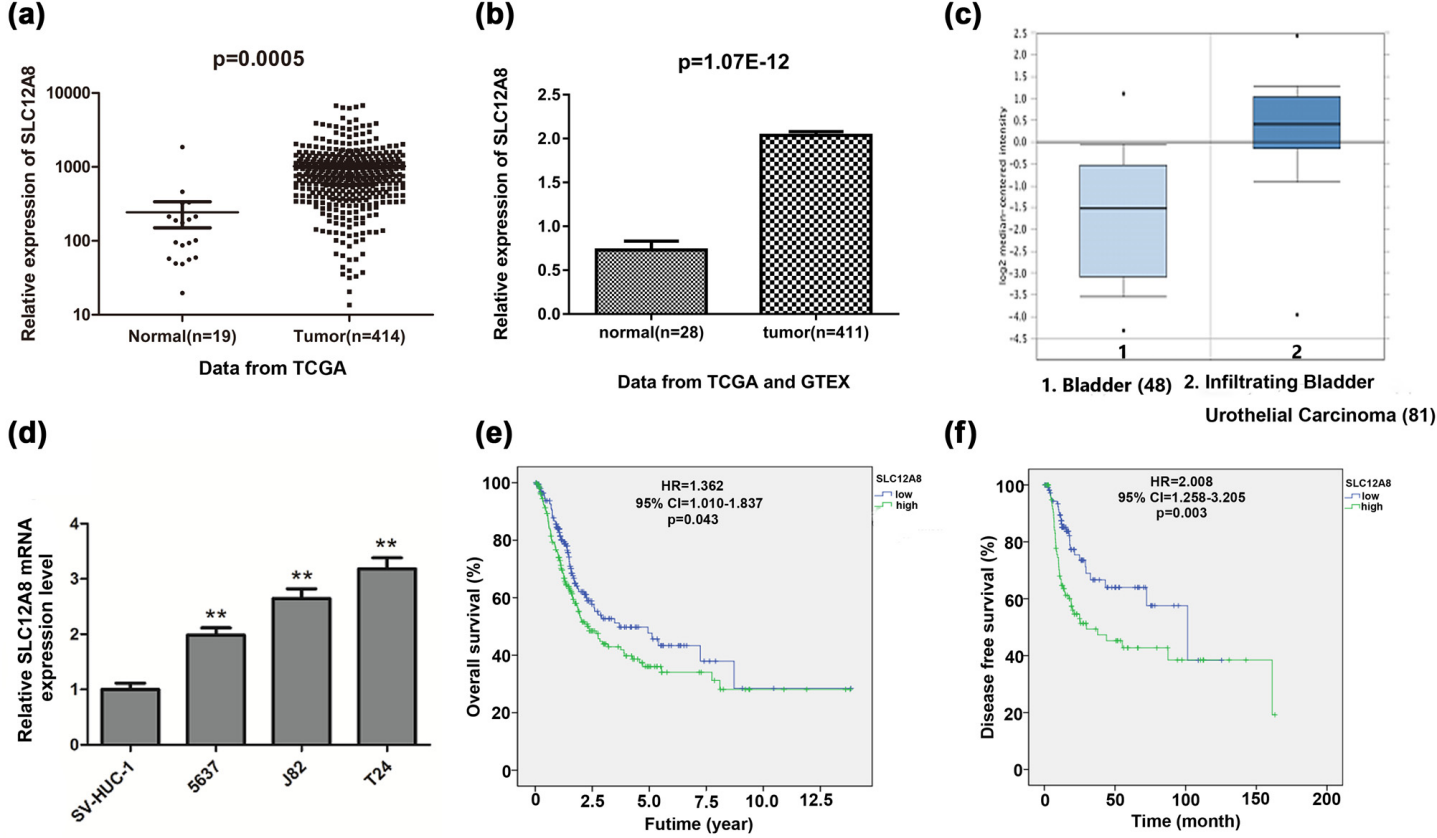
Expression of SLC12A8 in bladder cancer and its prognostic value. (a) SLC12A8 expression level was remarkably higher in bladder cancer tissues (*n* = 414) than that in normal tissues (*n* = 19) based on data from the TCGA database, *p* = 0.0005. (b) Data from TCGA and GTE_*X*_ showed that SLC12A8 was significantly upregulated in bladder cancer samples (*n* = 411) than that in normal samples (*n* = 28), *p* = 1.07 × 10^−12^. (c) SLC12A8 expression was meaningfully higher in infiltrating bladder urothelial carcinoma (*n* = 81) compared with normal bladder (*n* = 48) in the Oncomine dataset, *p* < 0.0001. (d) SLC12A8 expression in (5637, J82, and T24) cell lines was higher than normal control cells (SV-HUC-1), **p < 0.01. (e) Kaplan–Meier analysis showed the bladder cancer patients’ survival with unequal SLC12A8 expression level, *p* = 0.043. (f) Kaplan–Meier analysis showed that high expression of SLC12A8 led to the worse disease-free survival in patients with bladder cancer, *p* = 0.003.

Rely on the TCGA database, the SLC12A8 expression level was demonstrated notably higher in bladder cancer tissue compared with normal tissue (*p* = 0.0005; Figure 1a). Moreover, data from TCGA and GTEx presented the similar result, and SLC12A8 was highly expressed in bladder cancer compared with the normal samples (*p* = 1.07 × 10^−12^; Figure 1b). Besides, the results from Sanchez-Carbayo Bladder 2 statistics from Oncomine database indicated that SLC12A8 expression was significantly enhanced in infiltrating bladder urothelial carcinoma compared with the normal bladder tissues, which was consistent with the aforementioned results (*p* < 0.0001, Figure 1c).

Afterward, the SLC12A8 expression levels in 5637, J82, and T24 cell lines and SV-HUC-1 cell line have been examined by applying qRT-PCR (Figure 1d). The outcomes suggested that the mRNA levels of SLC12A8 in 5637, J82, and T24 cell lines were notably higher compared with that in SV-HUC-1 cells (*p* < 0.01). From the results, the mRNA expression of SLC12A8 in T24 cells was the highest, while the expression value in 5637 cells was the lowest. Therefore, the T24 and 5637 cells were selected to carry out the subsequent SLC12A8 knockdown and upregulation experiments, respectively.

### 3.2 High SLC12A8 expression correlated with poor prognosis of bladder cancer

The correlation between SLC12A8 expression and clinicopathological features in bladder cancer patients is presented in Table 1. The bladder cancer patients were divided into SLC12A8 high expression group (*n* = 202) and SLC12A8 low expression group (*n* = 203) according to the median value of SLC12A8 expression. Table 1 demonstrates that the SLC12A8 expression was correlated with age (*p* ≤ 0.001), pathological stage (*p* ≤ 0.001), T stage (*p* ≤ 0.001), and N stage (*p* ≤ 0.001) of bladder cancer patients. However, the correlations between SLC12A8 expression and the gender and M stage were not significant.

**Table 1:** Correlation between SLC12A8 expression and clinical features of bladder cancer

Characteristics	Expression of SLC12A8		*p* value
	Low	High	*p* < 0.05
**Age**			≤0.001*
<60	61	26	
≥60	142	176	
**Gender**			0.350
Female	49	57	
Male	154	145	
**Pathologic stage**			≤0.001*
I + II	89	41	
III + IV	113	160	
**Pathologic-T**			≤0.001*
T1 + T2	76	45	
T3 + T4	105	146	
**Pathologic-N**			≤0.001*
N0	135	100	
N1	47	81	
**Pathologic-M**			0.059
M0	114	81	
M1	3	8	

*p < 0.05; T, tumor; N, lymph nodes; M, metastasis.

In addition, to explore whether SLC12A8 expression levels were associated with prognosis of bladder cancer, the Kaplan–Maier analysis and log-rank comparison were carried out. The results demonstrated that higher SLC12A8 expression level was correlated with shorter OS in bladder cancer patients (*p* = 0.043, Figure 1e). Moreover, higher SLC12A8 expression level led to the worse disease-free survival (*p* = 0.003, Figure 1f).

The Cox regression analysis results showed that the SLC12A8 expression, Clinical stage, T stage, lymph node, metastasis, and age were notably correlated with OS of bladder cancer patients. Besides, pathologic-N could be used as an independent prognostic factor of bladder cancer by the multivariate analysis (Table 2; *p* = 0.013, HR = 2.034, 95% CI: 1.165–3.552).

**Table 2:** COX regression analysis of univariate and multivariate factor of bladder cancer

Variables	Univariate analysis			Multivariate analysis		
	*p* value	HR	95% CI	*p* value	HR	95% CI
SLC12A8 expression (high/low)	0.043*	1.362	1.010–1.837	0.473	1.219	0.710–2.093
Clinical stage (I + II/III + IV)	≤0.001*	2.221	1.533–3.216	0.337	0.501	0.122–2.053
Pathologic-T (T1 + T2/T3 + T4)	≤0.001*	2.140	1.472–3.111	0.105	2.881	0.802–10.352
Pathologic-M (M0/M1)	0.002*	1.817	1.256–2.629	0.669	1.113	0.683–1.813
Pathologic-N (N0/N1 + N2 + N3)	≤0.001*	2.267	1.656–3.105	0.013*	2.034	1.165–3.552
Age (<60/≥60)	0.002*	1.996	1.288–3.093	0.591	1.221	0.589–2.533
Gender (female/male)	0.439	0.880	0.635–1.218			

*p < 0.05; HR: hazard ratio; M: metastasis; N: lymph nodes; T: tumor.

### 3.3 SLC12A8 knockdown/upregulation efficiency determined by qRT-PCR and western blot analysis

**Figure 2:**
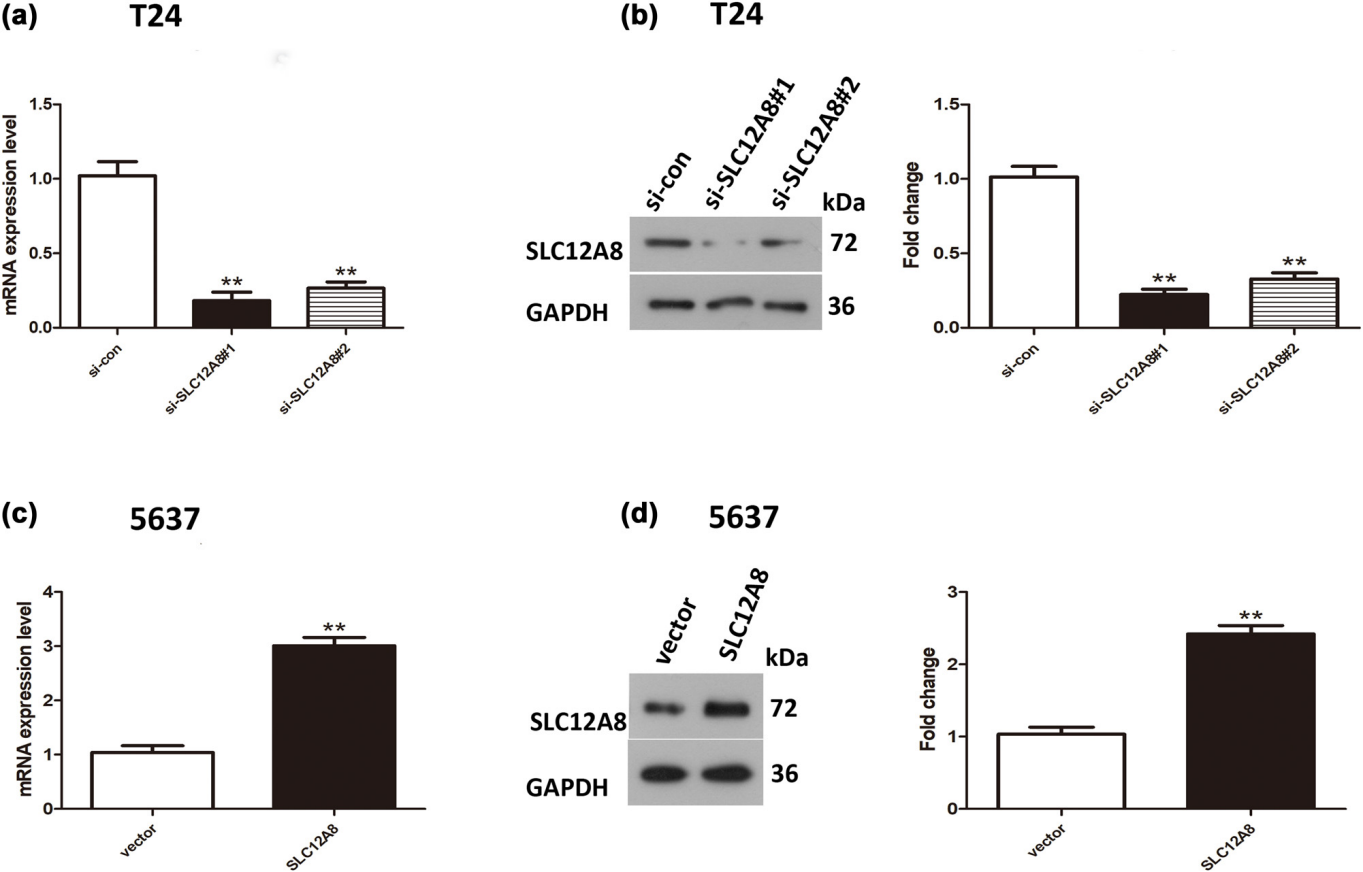
Expression of SLC12A8 mRNA and protein in bladder cancer cells transfected with si-SLC12A8 or pcDNA3.1-SLC12A8. (a) The SLC12A8 mRNA expression levels were decreased in T24 cells after transfection with si-SLC12A8#1 or si-SLC12A8#2, **p < 0.01. (b) The SLC12A8 protein expression was decreased in T24 cells with depleted SLC12A8, **p < 0.01. (c) The mRNA-SLC12A8 expression level was remarkably increased in 5,637 cells with upregulated SLC12A8, **p < 0.01. (d) The SLC12A8 protein expression was notably higher in 5,637 cells with upregulated SLC12A8, **p < 0.01.

In brief, human bladder cancer T24 cells were transfected with si-SLC12A8#1 and si-SLC12A8#2, while 5637 cells were transfected with pcDNA3.1-SLC12A8. The SLC12A8 mRNA and protein levels in T24 and 5637 cells were detected by qRT-PCR and western blot. The results of qRT-PCR suggested that mRNA-SLC12A8 level in T24 cells with depleted SLC12A8 was evidently lower than that in the control group (*p* < 0.01) (Figure 2a). The inhibitory effect was best in the si-SLC12A8#1 group, and the relative expression of mRNA was 17.84% of the control group. The outcomes of western blot revealed that the lower SLC12A8 protein expression was presented in si-SLC12A8#1 group (*p* < 0.01) (Figure 2b). For the accuracy of the inference experiment, the following loss of function assays was performed using both si-SLC12A8#1 and si-SLC12A8#2. However, the mRNA and protein expression levels of SLC12A8 were notably promoted in 5637 cells transfected with pcDNA3.1-SLC12A8 (Figure 2c and d).

### 3.4 Knockdown/upregulation of SLC12A8 affected the growth, invasion, and migration of bladder cancer cells

**Figure 3:**
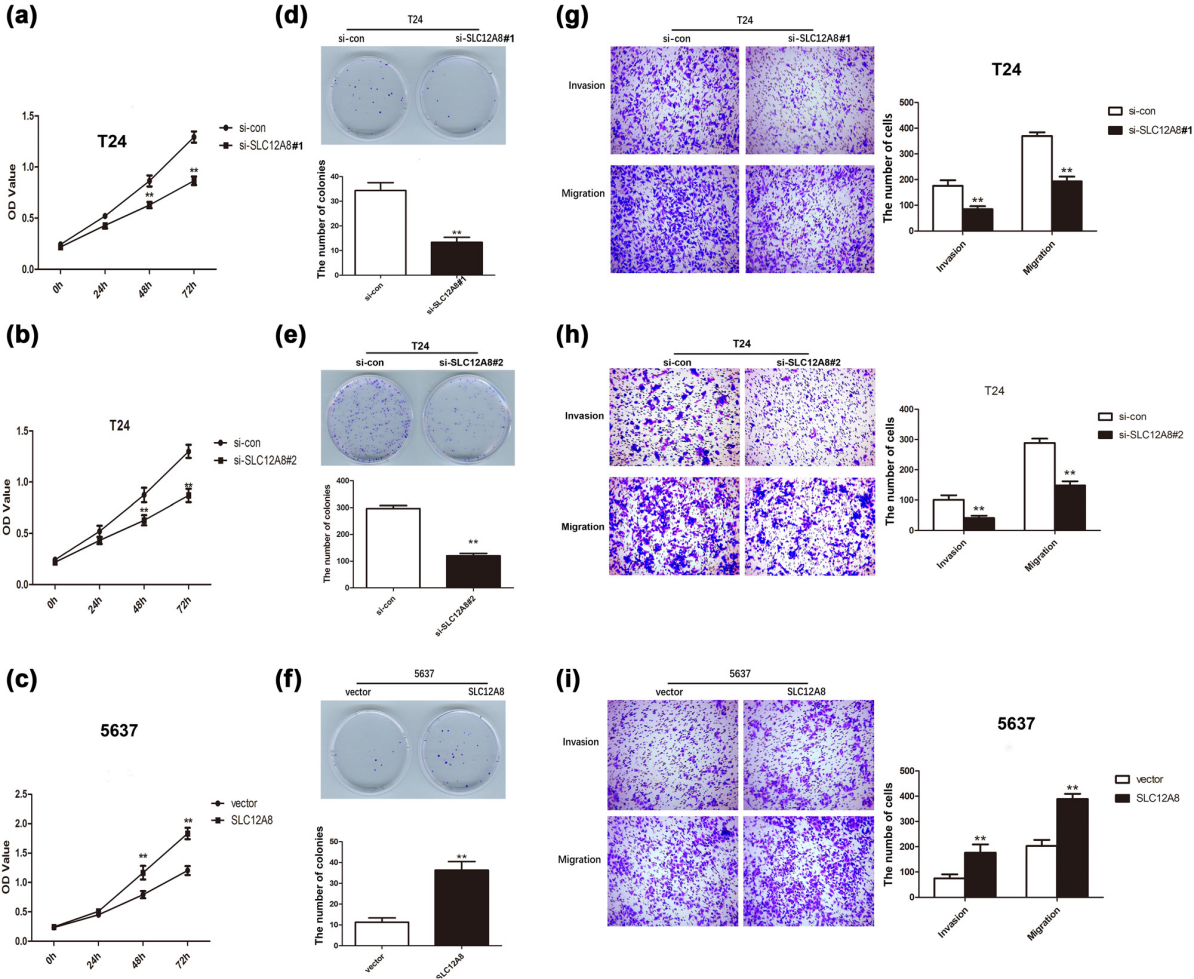
SLC12A8 knockdown or upregulation affected the growth of bladder cancer cells. (a) The proliferation of T24 cells with downregulated SLC12A8 detected by CCK8, **p < 0.01. (b) The proliferation of T24 cells was examined by CCK8 after knockdown of SLC12A8 by si-SLC12A8#2, **p < 0.01. (c) The proliferation of 5,637 cells with upregulation of SLC12A8 detected by CCK8, **p < 0.01. (d) The plate cloning experiments showed that the proliferation rate of T24 cells in the si-SLC12A8#1 group was remarkably reduced compared with the si-con group, **p < 0.01. (e) Knockdown of SLC12A8 by si-SLC12A8#2 reduced the colony formation ability of T24 cells, **p < 0.01. (f) The plate cloning experiments showed that the proliferation rate of 5,637 cells in the pcDNA3.1-SLC12A8 group was notably promoted compared with vector group, **p < 0.01. (g) Knockdown of SLC12A8 by si-SLC12A8#1 repressed the invasion and migration of T24 cells, **p < 0.01. (h) The invasion and migration abilities of T24 cells were inhibited by si-SLC12A8#2 treatment, **p < 0.01. (i) Upregulation of SLC12A8 enhanced the invasion and migration of 5,637 cells, **p < 0.01.

To further explore the effect of SLC12A8 on the proliferation and motility of bladder cancer cells, CCK8 and colony formation assays were carried out in T24 and 5673 cells, which transfected with si-SLC12A8#1 and si-SLC12A8#2 or pcDNA3.1-SLC12A8, respectively. The results of the CCK8 assay indicated that the proliferative ability of T24 cells with silenced SLC12A8 by si-SLC12A8#1 or si-SLC12A8#2 was notably decreased (*p* < 0.01) (Figure 3a and b). However, it was showed that the viability of 5,637 cells was significantly promoted after upregulation of SLC12A8 (*p* < 0.01) (Figure 3c). The outcomes of plate cloning experiment showed that compared with the control group, the proliferation rate of the T24 cells was significantly reduced after knockdown of SLC12A8 by si-SLC12A8#1 or si-SLC12A8#2 (Figure 3d–e) (*p* < 0.01). Figure 3f showed that the results of plate cloning experiments indicated that the number of colonies in 5,637 cells group (36.33 ± 7.09) increased compared with the control group (11.33 ± 3.51) (*p* < 0.01). The above experimental data demonstrated that SLC12A8 upregulation could notably promote the proliferative capacity of bladder cancer cells.

Next, the Transwell assays were used to detect the association between SLC12A8 expression and bladder cancer metastasis. The outcomes showed that the invasive ability of the si-SLC12A8#1 group was 48.38% of the control group (*p* < 0.01), while the migratory ability was 52.26% of the control group (*p* < 0.01, Figure 3g). Similarly, the invasive (40.73%) and migratory (51.27%) abilities of T24 cells were reduced after knockdown of SLC12A8 by si-SLC12A8#2 treatment (*p* < 0.01, Figure 3h). The aforementioned results indicated that the knockdown of SLC12A8 can reduce the invasive and migratory capacities of bladder cancer cells. On the contrast, the number of transmembrane cells in pcDNA3.1-SLC12A8 group was notably increased. The invasive ability was 2.37 times of the control group (*p* < 0.01), while the migratory ability was 1.91 times of the control group (*p* < 0.01) (Figure 3i), illustrating that the invasive and migratory capacities of bladder cancer cells were enhanced by SLC12A8 upregulation.

### 3.5 EMT mediated the function of SLC12A8 in regulating bladder cancer

**Figure 4:**
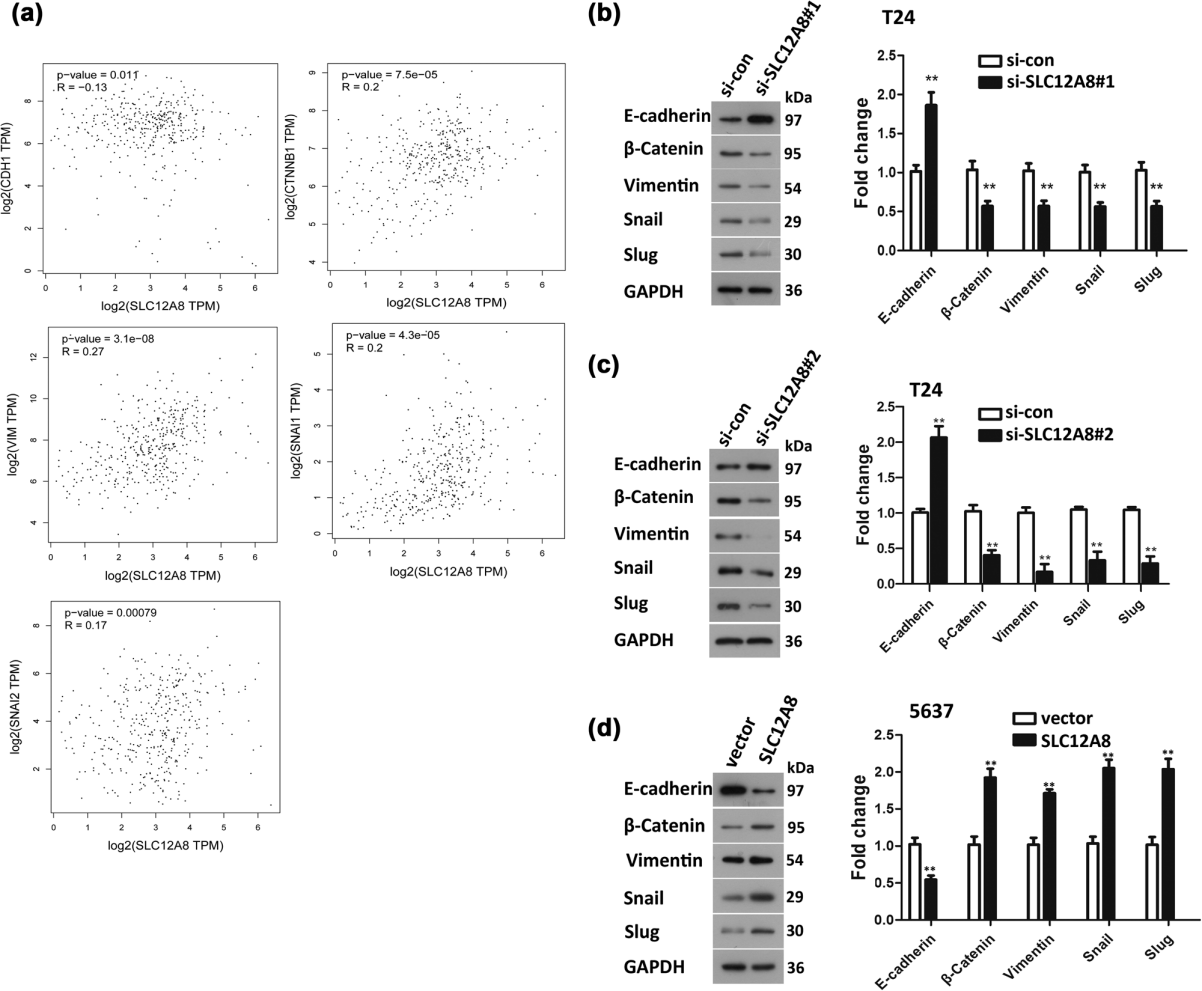
EMT mediated the function of SLC12A8 in regulating bladder cancer. (a) The correlation between SLC12A8 and EMT markers was analyzed in GEPIA website by Pearson correlation coefficient. (b and c) The expression of EMT protein markers in T24 cells was detected by western blotting after depletion of SLC12A8 by si-SLC12A8#1 or si-SLC12A8#2, **p < 0.01. (d) The expression of EMT protein markers in 5637 cells was examined by western blotting with upregulation of SLC12A8, **p < 0.01.

The correlation between SLC12A8 and EMT marker including CDH1 (E-cadherin), CTNNB1 (β-catenin), VIM (vimentin), SNAIL1 (snail), and SNAIL2 (slug) was analyzed in GEPIA website by the Pearson correlation coefficient (Figure 4a). The data showed that SLC12A8 expression was negatively correlated with E-cadherin and positively correlated with β-catenin, vimentin, snail, and slug. The western blot evidenced the aforementioned results: β-catenin, vimentin, snail, and slug protein were decreased, and E-cadherin protein was increased when SLC12A8 was knocked down by si-SLC12A8#1 or si-SLC12A8#2 in T24 cells (Figure 4b and c), while the result was opposite when SLC12A8 was upregulated in 5,637 cells (Figure 4d). The aforementioned outcomes revealed that SLC12A8 upregulation could remarkably promote the EMT process of 5,637 cells, while the EMT in T24 cells was notably inhibited when SLC12A8 was depleted.

## 4 Discussion

As one of the common malignant tumors, bladder cancer has a high recurrence rate and seriously affects people’s live. Currently, the treatment of bladder cancer is still based on surgical resection, but the prognosis is poor [23]. Early diagnosis and timely treatment can improve the survival rate and effectively avoid local recurrence and distant metastasis, which is a key factor to improve the prognosis of patients with this disease [22]. Up to now, the mechanism of bladder cancer is not very clear, and its occurrence and development are affected by a variety of genetic changes [24]. The mRNA expression profile in public databases provides a new way for us to investigate tumorigenesis [25]. In this study, SLC12A8, a differentially expressed gene, was screened out using the TCGA database, and the expression level of SLC12A8 in bladder cancer and its correlation with pathological and prognosis were revealed, which provide the foundation and ideas for further study of its mechanism in bladder cancer.

SLC12A8 is thought to be a candidate for psoriasis susceptibility [26]. Kim et al. found that SLC12A8 was correlated with a shorter overall survival of breast cancer patients, probably because the variation of SLC12A8 may be involved in the transportation of chemotherapeutic drugs, leading to a worse prognosis [19]. However, the expression of SLC12A8 in bladder cancer and its mechanism of action have not been reported. Up to now, this is the first study reporting that upregulation of SLC12A8 promoted the development and metastasis of the bladder cancer and correlated with poor prognosis. In our study, we found that SLC12A8 is highly expressed in tumor tissues and promotes proliferation, invasion, and migration of cancer cells, which confirms that SLC12A8 affects the biological functions of bladder cancer cells and insinuates that SLC12A8 may play an oncogenic role in the progression of bladder cancer.

EMT is a physiological process existing in human tissues and participates in embryonic development and tissue growth, associating with diseases such as tissue fibrosis and tumors [27]. Tumor metastasis is closely related to EMT, and EMT occurs before tumor metastasis, so EMT is considered to be an early marker of tumor metastasis [28,29]. It is found that EMT plays a determinant role in metastatic potentiality and the beginning period of cancer metastasis, by promoting the invasion and proliferation of human cancers, including bladder cancer [30,31,32]. Luo et al. reported that EMT is a significant mechanism that increased the invasive capacity of bladder cancer cells [33]. E-Cadherin, β-catenin, vimentin, snail, and slug are pivotal protein markers of EMT, and their expressions indicate changes of the EMT process [34,35]. Our research indicated that SLC12A8 upregulation could notably promote the EMT process in bladder cancer, suggesting that SLC12A8 might promote the malignant development of tumor by regulating the EMT process.

Reports about the specific function of SLC12A8 on cancer cells or normal cells proliferation and EMT were limited. Via reviewing the published articles, we observed that SLC12A1, a member of SLC12A family, was a positive regulator of WNK/ERK5 pathway, which can promote cell proliferation and survival [11]. Moreover, suppressing SLC12A1 might inhibit proliferation-related genes like Cyclin D1, a key proliferation checkpoint [36]. According to the aforementioned observations, we speculated that SLC12A8 may promote the proliferation of tumor cells partly through the WNK/ERK5 pathway, which needed to be verified in the future.

Experiments *in vivo* and externally clinical cohort validation are urgently needed to verify our present data. How SLC12A8 affects the progression of bladder cancer also requires further exploration, which is the next research topic we will carry out.

## 5 Conclusion

Our research demonstrated that SLC12A8 was closely related to the malignant degree of bladder cancer and affect the patients’ survival. Therefore, SLC12A8 is expected to be an indicator to evaluate the prognosis of bladder cancer patients as well as an effective therapeutic target for bladder cancer treatment.
